# Impact and consequences of intensive chemotherapy on intestinal barrier and microbiota in acute myeloid leukemia: the role of mucosal strengthening

**DOI:** 10.1080/19490976.2020.1800897

**Published:** 2020-09-06

**Authors:** Thomas Hueso, Kenneth Ekpe, Camille Mayeur, Anna Gatse, Marie Joncquel-Chevallier Curt, Guillaume Gricourt, Christophe Rodriguez, Charles Burdet, Guillaume Ulmann, Christel Neut, Salah-Eddine Amini, Patricia Lepage, Bruno Raynard, Christophe Willekens, Jean-Baptiste Micol, Stéphane De Botton, Ibrahim Yakoub-Agha, Frédéric Gottrand, Jean-Luc Desseyn, Muriel Thomas, Paul-Louis Woerther, David Seguy

**Affiliations:** a Univ. Lille, Inserm, CHU Lille, U1286 - INFINITE - Institute for Translational Research in Inflammation, Lille, France; b Micalis Institute, INRAE, AgroParisTech, Université Paris-Saclay, Jouy-en-Josas, France; c Department of Biochemistry, CHU Lille, Lille, France; d NGS Platform, IMRB, CHU Henri Mondor, Créteil, France; e Institut Mondor de Recherche Biomédicale, Inserm U955, Créteil, France; f School of Medicine, EA3964 University of Paris Diderot, Sorbonne Paris Cité, Paris, France; g Department of Biochemistry, Cochin Hospital – HUPC, Paris, France; h Nutrition Department, Gustave Roussy Cancer Centre, F-94805, Villejuif, France; i Hematology Departement, Gustave Roussy Cancer Centre, F-94805, Villejuif, France; j Allogeneic Stem Cell Department, CHU Lille, Lille, France; k Department of Microbiology and Infection Control, Henri-Mondor Hospital, Créteil, France; l EA 7380 Dynamyc, EnvA, UPEC, Paris-Est University, Créteil, France; m Nutrition Unit, CHU Lille, Lille, France

**Keywords:** Intestinal barrier, microbiota, chemotherapy, acute leukemia, mucus

## Abstract

Induction chemotherapy (7 + 3 regimen) remains the gold standard for patients with acute myeloid leukemia (AML) but is responsible for gut damage leading to several complications such as bloodstream infection (BSI). We aimed to investigate the impact of induction chemotherapy on the intestinal barrier of patients with AML and in wild-type mice. Next, we assessed the potential benefit of strengthening the mucosal barrier in transgenic mice releasing a recombinant protein able to reinforce the mucus layer (Tg222). In patients, we observed a decrease of plasma citrulline, which is a marker of the functional enterocyte mass, of short-chain fatty acids and of fecal bacterial load, except for *Escherichia coli* and *Enterococcus* spp., which became dominant. Both the α and β-diversities of fecal microbiota decreased. In wild-type mice, citrulline levels decreased under chemotherapy along with an increase of *E. coli* and *Enterococcus* spp load associated with concomitant histologic impairment. By comparison with wild-type mice, Tg222 mice, 3 days after completing chemotherapy, had higher citrulline levels, a faster healing epithelium, and preserved α-diversity of their intestinal microbiota. This was associated with reduced bacterial translocations. Our results highlight the intestinal damage and the dysbiosis induced by the 7 + 3 regimen. As a proof of concept, our transgenic model suggests that strengthening the intestinal barrier is a promising approach to limit BSI and improve AML patients’ outcome.

## Introduction

Induction chemotherapy that combines seven days of cytarabine and three days of anthracycline (7 + 3 regimen) remains the standard of care for patients with acute myeloid leukemia (AML), with a 70 to 80% complete remission rate.^1^ Subsequently, most patients undergo consolidation and conditioning chemotherapy preceding allogeneic stem cell transplantation (allo-SCT). During these treatments, several complications may mitigate the prognosis of AML, such as bloodstream infections (BSI), relapse of the hematological disease, or acute graft-versus-host disease (GvHD) after allo-SCT. Because of their toxicity, chemotherapies are responsible for the intestinal barrier failure that promotes BSI usually caused by gram-negative bacteria of the digestive tract.^2,3^ Furthermore, the widespread use of antibiotics enhances the dissemination of multidrug-resistant bacteria, raising the health costs, and increasing the infection-related mortality rate.^4^

The intestinal barrier is composed of three different components: the microbiota, the mucus layer, and the epithelial layer. Mucus interfaces the microbiota and mucosa allowing reciprocal and dynamic interactions that maintain gut homeostasis and act as physical, biochemical, and biological defenses against aggressions and infections.^5–7^ Mucus is essentially composed of water (>90%). Gel-forming mucins such as MUC2 represent the main organic component of the ileal and colonic mucus gel.^8^ The highly conserved mucin CYS domain found in two copies in MUC2 acts as a natural crosslinker of MUC2 polymers that reinforces the mucus layer and mainly provides resistance against pathogen colonization.^9^ Our team generated a transgenic (Tg) mouse (line Tg222) able to release a string of a higher number of CYS domains (n = 12) in the intestinal lumen that strengthens the mucus barrier. We have previously demonstrated that the colonic mucus of the Tg mice is more protective against bacterial translocation and houses a higher abundance of *Lactobacillus* spp in comparison to their wild-type (Wt) littermates. Such Tg mice are less sensitive to dextran sodium sulfate-induced colitis and to bacterial translocation.^10^

The aim of this translational study was to describe the intestinal barrier, including the microbiota, mucus, and epithelium in both AML patients and preclinical mouse models. The composition of microbiota has been assessed before, during, and after a 7 + 3 regimen in AML patients. Because intestinal biopsies cannot be routinely performed in such patients, we assessed gut impairment by measuring plasma concentrations of citrulline, a marker of functional enterocyte mass, and fecal short-chain fatty acids (SCFA) produced by intestinal microbiota, which are essential for maintaining colonic trophicity.^11–13^ To further determine the impact of chemotherapy on intestinal impairment, we studied a murine model mimicking induction chemotherapy for AML without antibiotics. Finally, we challenged the Tg222 mice to investigate the potential effect of strengthening the mucosal barrier during induction chemotherapy.

## Results

### Human

#### Patients’ outcome

**Figure 1.**
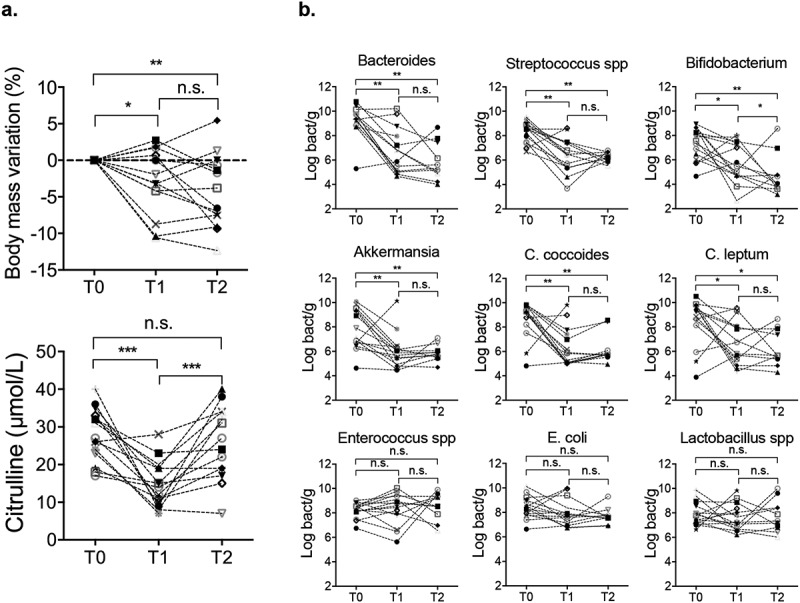
(a) Body mass variation and plasma citrulline level in patients before induction (T0), during aplasia (T1) and after hematological recovery (T2). (b) Quantitative PCR for aerotolerant and anaerobic bacteria in patients’ feces. *Bacteroides, Bifidobacterium, Akkermansia spp, C. coccoides and C. leptum* represented dominant groups. *Streptococcus spp, Enterococcus spp, E. coli* and *Lactobacillus spp* represented sub-dominant groups. *p* < .05 is considered significant, n.s.: not significant. *p < .05; **p < .01; ***p < .001.

Initial characteristics of the 15 patients enrolled are detailed in Table 1. All patients had a neutropenic fever and received broad-spectrum antibiotics during the 7 + 3 regimen. BSI occurred in 47% (7/15) of patients and was mostly due to *E. coli* (4/7). One patient died of a septic shock caused by refractory candidemia. There was a significant weight loss (expressed in percent of the weight at T0) at T1 (−2.6% [−5.7; 0.9]; *p* = .027) and T2 (−5.2% [−7.9; −0.5]; *p* = .006). Compared with T0 (29 [22–33] µmol/L), citrulline levels decreased significantly at T1 (14 [10–19] µmo/L; *p* = .0002), and reached near normal values at T2 (25 [18–34] µmol/L; *p* = .412) (Figure 1a). Over 70% of the patients (11/15) were in remission after induction chemotherapy (Figure 1a).

**Table 1.** Patient characteristics.

Patient characteristics	
**Age, median [range]**	54 [35–74]
**Sex, n (%)**	
Female	6 (40)
Male	9 (60)
**AML, n (%)**	
De novo	12 (80)
Secondary	3 (20)
**BMI at admission, median [range]**	25.3 [17.3–31]
**Induction chemotherapy, n (%)**	
AraC + Ida *	7 (47)
AraC + Dauno ^¥^	8 (53)
**Time of assessment^¶^, median [range]**	
T0	−2 [−12-0]
T1	12 [8–18]
T2	24 [18–35]
**ATB during induction phase, n (%)**	15 (100)
Tazocilline	10 (67)
Imipenem	9 (60)
Cefepime	5 (33)
Vancomycine	3 (20)
**During of ATB administration, day [range]**	28 [15–43]
**During of neutropenia, day [range]**	20 [9–35]
**Neutropenic fever, n (%)**	15 (100)
**Documented BSI^§^, n (%)**	7 (47)
*Escherichia coli*	4 (26)
*Enteroccocus spp*	1 (7)
*Klebsiella pneumoniae*	1 (7)
*Closdirium perfringens*	1 (7)
**Remission after induction, n (%)**	11 (73)

*Aracytine 200 mg/m^2^ from d1 to d7 and Idarubicin 12 mg/m^2^ from d1 to d3. ¥Aracytine 200 mg/m^2^ from d1 to d7 and Daunorubicin 60 mg/m^2^ from d1 to d3. ¶ Time of assessment considering the delay from the initiation of chemotherapy: T0: before induction, T1: during aplastic period, and T2: after hematological recovery phase. § BSI: blood stream infection documented with blood culture. AML: acute myeloid leukemia; Ida: idarubicin; AraC: aracytine; Dauno: Daunorubicin; BMI: body mass index; ATB: antibiotics.

#### Dysbiosis

The bacterial load of the feces assessed by qPCR decreased between T0 and T1 (−0. 8 log bacteria/g; *p* = .004) and remained low at T2 (−1 log bacteria/g; *p* = .05). The overall decrease observed from T0 to T1 affected the following groups: *Bacteroides* spp., *Streptococcus* spp., *Bifidobacterium* spp., *Akkermansia, C. coccoides, C. leptum* with some individual variations. The bacterial load of dominant groups was not different between T1 and T2. The load of sub-dominant groups such as *Enterococcus* spp., *E. coli* and *Lactobacillus* spp. remained unchanged throughout the follow-up (Figure 1b).

**Figure 2.**
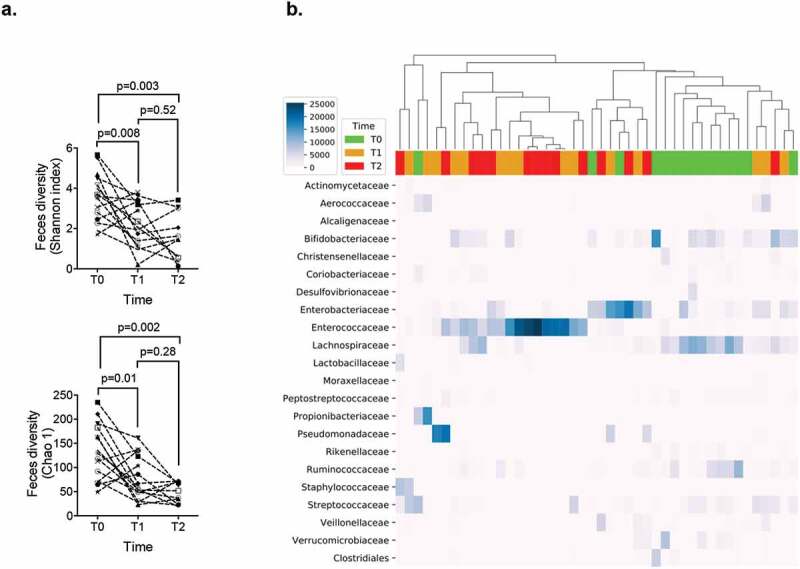
(a) Alpha-diversity in patients’ stool microbiome, represented by the Shannon index and Chao-1 index, before induction (T0), during aplasia (T1) and after hematological recovery (T2). *p* < .05 is considered significant. (b) A subset of OTUs, grouped by family, with a raw count at least of 300 was kept. Bray-Curtis distance and UPGMA algorithm were used to perform pairwise distance between samples and hierarchical clustering, respectively. The heatmap highlighted a clustering of two groups dominated by *Bifidobacteriaceae, Lachnospiraceae* and *Ruminococcaceae* at T0 progressively replaced by *Enterococcaceae or Enterobacteriaceae* at T1 and T2.

In next generation sequencing analyzes of human stool microbiota, alpha-diversity, assessed by the Shannon index, decreased between T0 (3.6 [2.45–4.5]) and both T1 (2.05 [1.14 − 3.35]) (*p* = .008) and T2 (1.46 [0.55–3.02]) (*p* = .003), but not between T1 and T2. Similarly, the Chao-1 index, decreased between T0 (160 [72–183]) and both T1 (62 [49–123]) (*p* = .01) and T2 (52 [23–70]) (*p* = .002), but not between T1 and T2. The unweighted Unifrac beta-diversity highlighted a modification of microbiota between T0 and both T1 (*p* = .02) and T2 (*p* = .001), but not between T1 and T2 (*p* = .25) as depicted in Figure 2a.

Analysis of the heatmap presented in Figure 2b resulted in the identification of two major groups of microbiota patterns. The first group was mostly composed of T0 samples characterized by the dominance of commensal *Bifidobacteriaceae, Lachnospiraceae*, and *Ruminococcaceae*. The second group was exclusively composed of both T1 and T2 samples and was characterized by the dominance of *Enterococcaceae*. These patterns corresponded to the time-dependent quantitative stability of *E. coli* and *Enterococcus* observed with qPCR. In two samples where *Enterococcaceae* were less abundant, microbiota composition was dominated by *Pseudomonadaceae*.

Unweighted Unifrac principal component analysis showed the clustering of the two groups of samples (T0, T1, and T2) according to their composition (Fig. S1).

#### SCFA released

Compared with T0, we observed a significant decrease of almost all SCFA concentrations at T1 and T2. At T2, all SCFA significantly decreased (Table 2).

**Table 2.** Comparison of fermentative activity in feces.

Fermentative activity (μmol/g)	T0	T1	T2	p*	p**
Acetate	11.5 [4.8–21.8]	2.6 [1.8–9.9]	3.4 [2.3–11.4]	*0.02*	*0.04*
Propionate	4.7 [1.3–7.2]	0.3 [0.1–0.9]	0.1 [0.1–0.7]	*0.08*	*0.03*
Butyrate	1.9 [0.2–3.4]	0.1 [0–1.5]	0 [0–0.3]	*0.2*	*0.03*
Valerate	0.2 [0.1–1.1]	0 [0–0.2]	0 [0–0]	*0.04*	*0.004*
Caproate	0	0	0	*n.s.*	*n.s.*
Isocaproate	0.4 [0.1–0.8]	0 [0–0.6]	0 [0–0.2]	*0.1*	*0.05*
Isovalerate	0.4 [0.2–0.9]	0 [0–0.5]	0 [0–0.2]	*0.09*	*0.05*

Comparison of short-chain fatty acids concentration between T0 *vs*. T1* and T0 *vs*. T2**, *p* < 0.05 is considered significant, n.s.: not significant.

### Mice

#### Outcome of induction chemotherapy model

**Figure 3.**
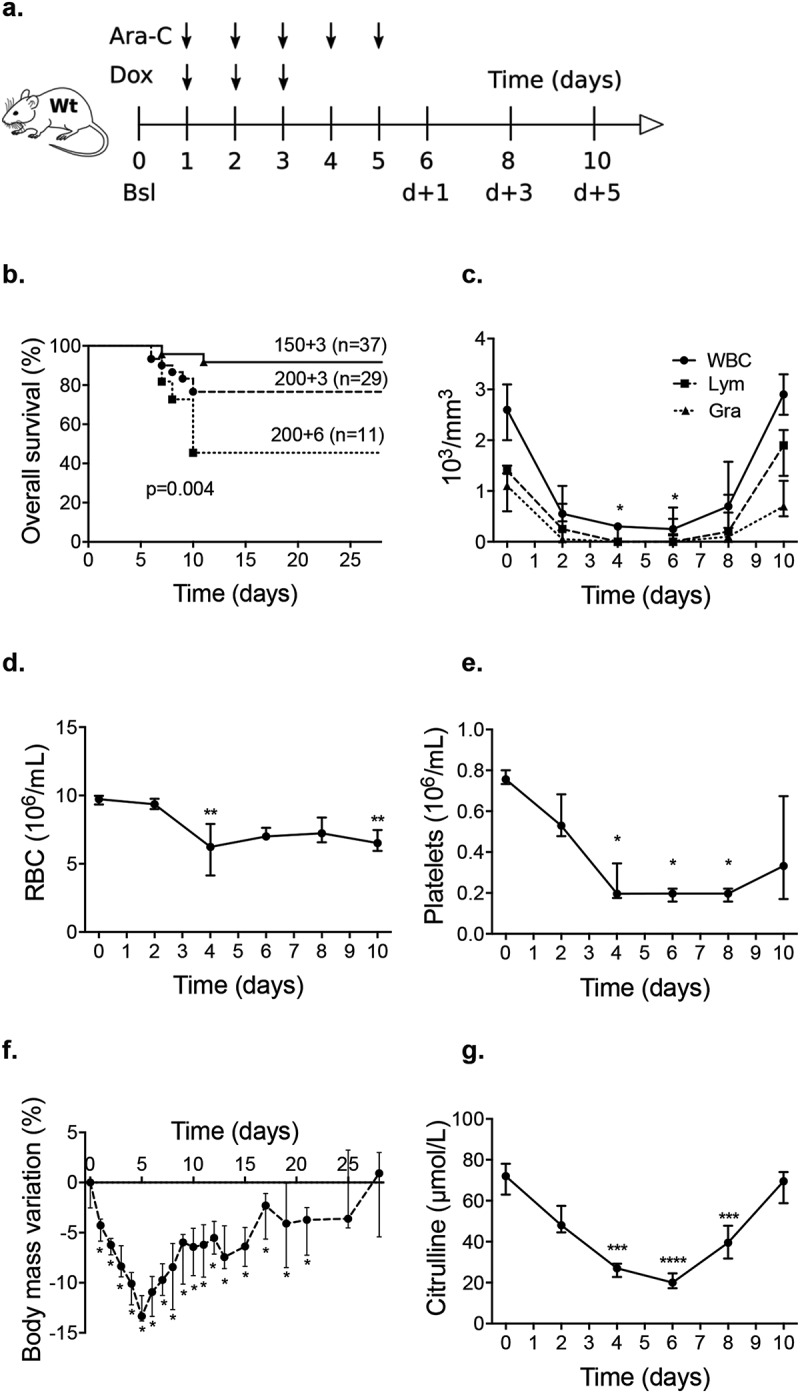
(a). Induction chemotherapy in a wild-type mouse model that consisted of a combined administration of aracytine (AraC) for five days and doxorubicin (Dox) for three days injected intraperitoneally. Baseline (Bsl) corresponded to the time before chemotherapy administration; d + 1, d + 3, and d + 5 corresponded to one day, three days and five days after the completion of chemotherapy. (b) Overall survival for different chemotherapies: AraC (5 days) + Dox (3 days). Triangle: AraC (150 mg/kg/d) + Dox (3 mg/kg/d); circle: AraC (200 mg/kg/d) + Dox (3 mg/kg/d); and square: AraC (200 mg/kg/d) + Dox (6 mg/kg/d). **c – g**. A model with AraC 150 mg/kg/d and Dox 3 mg/kg/d was elected resulting in decrease of blood count body weight and plasma citrulline level (n = 10 to 15 at each time). Wt: wild-type B6D2F1; WBC: white blood count; Lym: lymphocyte; Gra: granulocytes; RBC: red blood count. *p* < .05 is considered significant, * *p* < .05; **p < .01; ***p < .001.

Similarly to what was observed in patients, the mortality was low after completion of the induction chemotherapy. Mice experienced a transient decrease in both blood count and body mass, which normalized within 1 week and 3 weeks, respectively (Figure 3a–f). Citrulline level also decreased throughout chemotherapy administration until d + 3 and returned to normal at d + 5 following its completion (Figure 3g).

**Figure 4.**
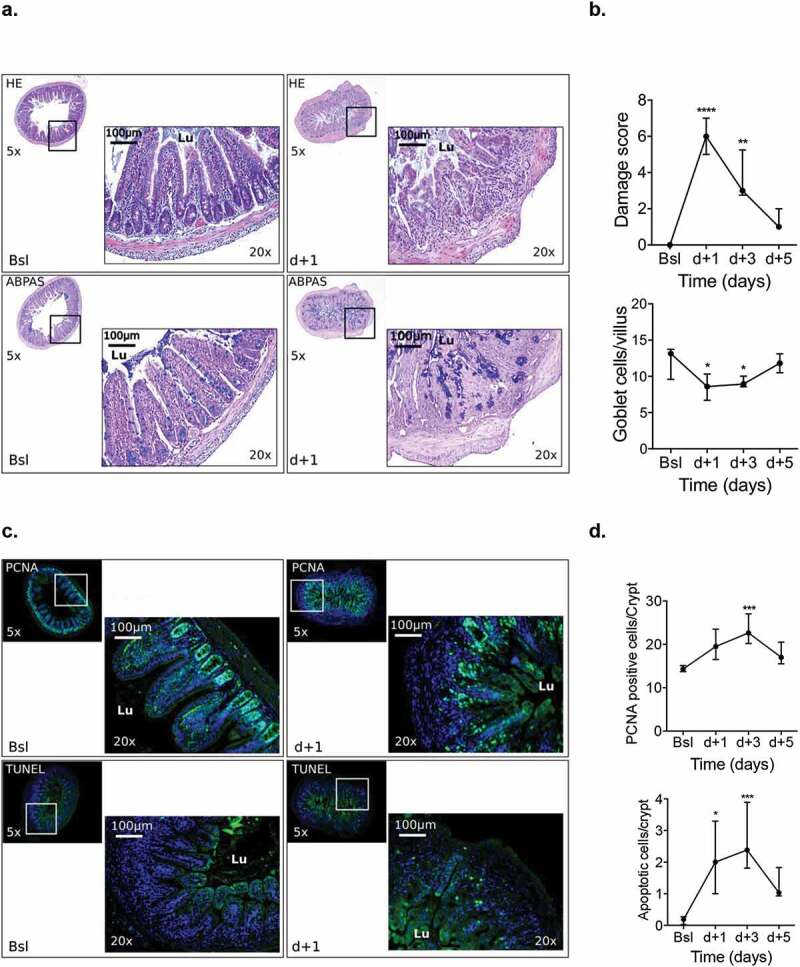
(a). Histological analyses of terminal ileum mucosa after HE and ABPAS staining, (b) Representative immunostaining of PCNA and apoptosis along the ileal villus-crypt axis (in green) at d + 1 and d + 3 and d + 5. (n = 7 to 10). Other cells were counterstained with Hoechst 33258 (in blue). HE: Hematoxylin/eosin; ABPAS: Alcian blue/periodic-acid Schiff; PCNA: proliferating cell nuclear antigen; TUNEL: TDT-mediated dUTP-biotin nick end-labeling; Lu: lumen. *p* < .05 significant, *p < .05; **p < .01.

Compared with baseline (0 [0–0]), histological damage score of the terminal ileum mucosa increased at d + 1 (6 [5–7]) (*p* < .0001) and remained high until d + 3 (3 [3–5]) (*p* = .005), before returning to baseline values at d + 5 (1 [1–2]); *p* = .652). The number of goblet cells followed the same kinetic variation: compared to baseline (13 [11–14]), goblet cells decreased significantly at d + 1 (8 [7–10]; *p* = .019); d + 3 (9 [8–10]; *p* = .035) and progressively returned to normal values at d + 5 (12 [10–13]; *p* = .9). Similarly, compared to baseline, the number of both PCNA-positive (14 [14–15]) and apoptotic cells (0 [0–0]) rose at d + 1 (20 [17–23] and 2 [1–3]); (*p* = .1 and *p* = .02), and d + 3 (23 [20–27] and 2 [2–4]); (*p* = .002 and *p* = .0008), respectively, and then decreased to return to normal values at d + 5 (17 [15–20] and 1 [1–2]); (*p* = .686 and *p* = .249) (Figure 4a-d).

We observed a similar bacterial load in the terminal ileum, spleen, and liver at baseline and d + 3 in Wt. However, we identified exclusively *Lactobacillus* spp. at baseline, whereas aerotolerant species such as *E. coli* and/or *Enterococcus* spp., appeared at d + 3 (Fig. S2).

#### Outcome of reinforced mucus layer model

**Figure 5.**
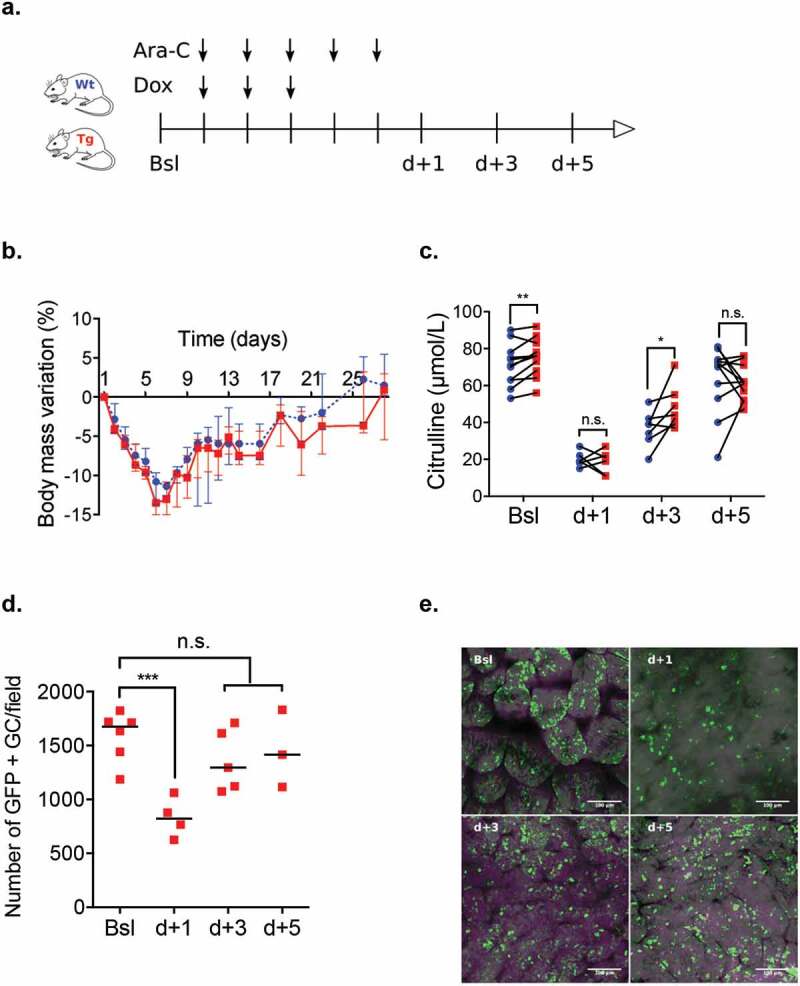
(a) Induction chemotherapy model (AraC (150 mg/kg.d) + Dox (3 mg/kg/d)) in Wt and Tg mice. (b – c) Comparison of weight loss and plasma citrulline level of Wt (blue circles) and Tg mice (red squares) at baseline (Bsl) and d + 1, d + 3 and d + 5 after chemotherapy completion. (d – e) Tracking of GFP-tagged transgene using epifluorescence microscopy (in green) in the ileal lumen of Tg mice from Bsl to d + 5. Ara-C: aracytine; Dox: doxorubicin; GC: Goblet cells;. *p* < .05 is considered significant, n.s., not significant. *p < .05; **p < .01.

Mouse weights remained comparable between Wt and Tg groups throughout induction chemotherapy (Figure 5a and b). However, at baseline, citrulline levels were higher in the Tg (76 [68–83] μmol/L) than in the Wt group (71 [63–78] μmol/L; *p* = .0017). Citrulline levels reached a nadir averaging at d + 1 in both groups but rose faster in Tg mice (44 [39–55] μmol/L vs. 39 [31–42] μmol/L; *p* = .046) at d + 3 (Figure 5c). In the latter, tracking of the GFP-tagged transgene by confocal microscopy showed a transient significant decrease of fluorescence at d + 1 that progressively returned to normal value at d + 5 (Figure 5d and e).

After induction chemotherapy, histological recovery was faster at d + 3 in Tg compared to Wt mice: a lower damage score (2 [1–2] vs. 3 [3–5]; *p* = .008) with higher villi, deeper crypt, and more goblet cells per villus and PCNA-positive cells per crypt. Increase of apoptotic cells, mainly localized in crypts, was similar in the two groups (Figure 6a-c).

**Figure 6.**
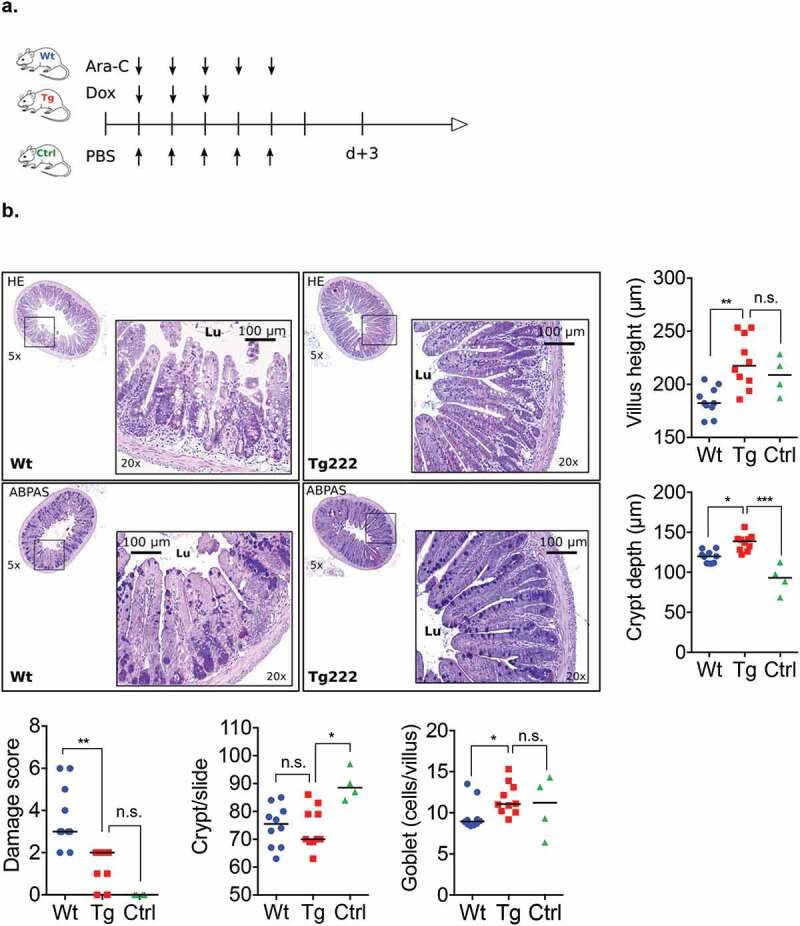
(a) Transgenic and wild type mice received chemotherapy regimen. Control group received PBS (included 2 Tg and 2 Wt mice). All mice were sacrificed at d + 3. (b-c) Histological analyses with HE and ABPAS staining and representative immunostaining with PCNA and apoptosis along the ileal villus-crypt axis (in green) at d + 3 after completion of induction chemotherapy. Other cells were counterstained with Hoechst 33258 (in blue).(d) Metabarcoding sequencing analyses of alpha diversity in ileum adherent microbiota at d + 3 in Wt and Tg mice and in chemotherapy and control groups. Ara-C: aracytine; Dox: doxorubicin; PBS: phosphate-buffered saline; HE: Hematoxylin/eosin; ABPAS: Alcian blue/periodic-acid Schiff; PCNA: proliferating cell nuclear antigen; TUNEL: TDT-mediated dUTP-biotin nick end-labeling; Lu: lumen. *p* < .05 is considered significant; n.s., not significant.

**Figure 6.**
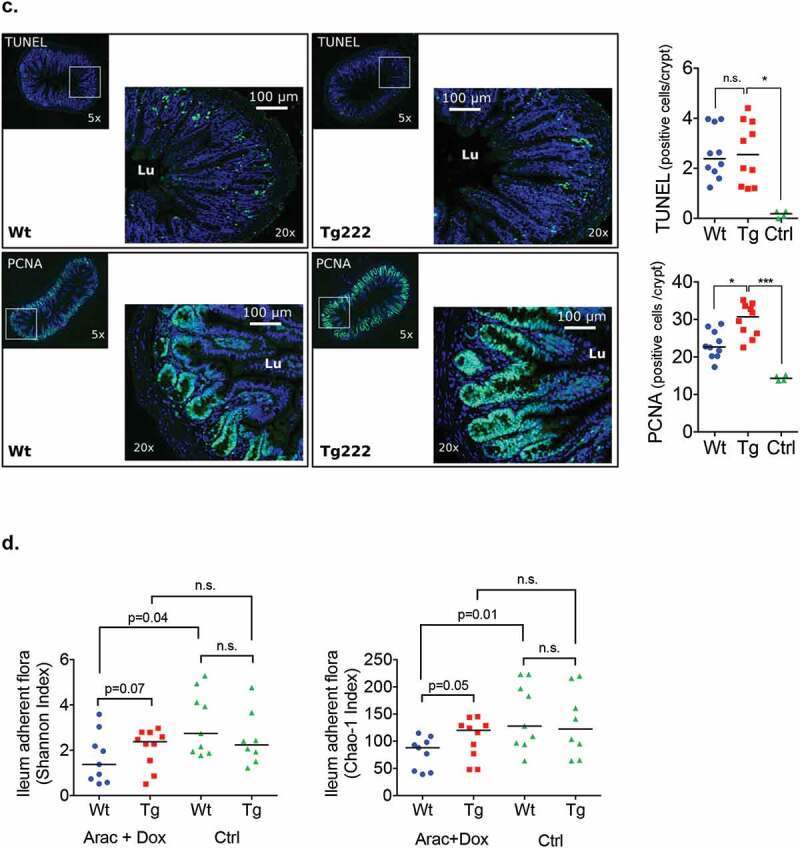
(Continued).

Quantitative PCR analysis of adherent flora of the terminal ileum revealed similar concentration of overall bacteria and *Lactobacillus* spp. *(L. murinus, L. reuteri, L. gasseri*) at baseline and d + 3 in both Tg and Wt groups. Concentration of *C. leptum* was also comparable at baseline between the two groups (6.42 [5.93–6.56] log bacteria/g vs. 6.39 [5.96–6.88] log bacteria/g; *p* = .81), but was higher in Tg group at d + 3 (6.44 [5.89–7.07] log bacteria/g vs. 5.41 [4.97–6.51] log bacteria/g; *p* = .02) (Fig. S3).

Results of alpha-diversity at d + 3 are detailed in Figure 6d. They were similar between Tg and Wt mice within the control group. Despite a trend of higher Shannon index (2.47 [1.9–2.8] vs. 1.38 [0.66–2.61]; *p* = .07), only the Chao-1 index was higher in Tg mice compared with Wt mice at d + 3 (124 [86–137] vs. 88 [43–104]; *p* = .05). In addition, while the indexes were lower in Wt than in control mice (1.38 [0.66–2.61] vs. 2.74 [1.91–4.53]; *p* = .04 and 88 [43–104] vs. 128 [96–210]; *p* = .01, respectively), they remained stable in Tg mice. With regards to the unweighted Unifrac beta-diversity at d + 3, no difference was observed between Tg and Wt mice in the control group (*p* = .44) and after chemotherapy (*p* = .84). Compared with the control group, unweighted Unifrac beta diversity showed a modified microbiota in the chemotherapy groups for both Wt (*p* = .011) and Tg mice (*p* = .043). Unweighted Unifrac principal component analysis evidenced the overall modification of the microbiota composition after chemotherapy in both Wt and Tg mice (Fig. S4).

**Figure 7.**
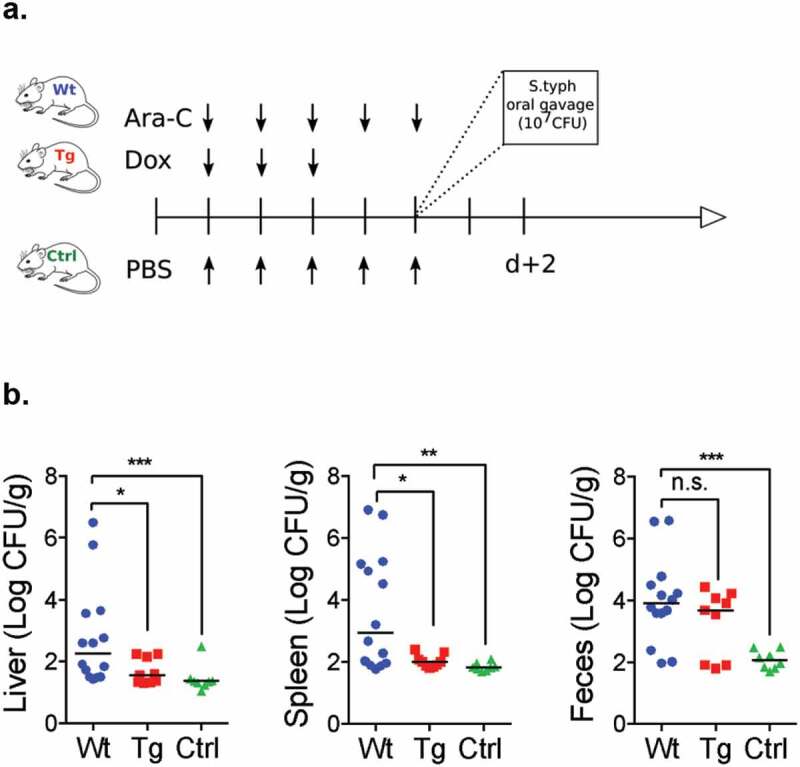
Oral gavage of 10^7^ CFU of *Salmonella* Typhimurium (*S*. Typhimurium) was conducted the last day of the chemotherapy regimen in Wt, Tg and controlled (Ctrl) mice. (b) Comparison of intestinal translocation of *S*. Typhimurium in Wt (blue circles), Tg (red squares) and Ctrl mice (green triangles) in liver spleen and feces at d + 2 after oral gavage. Ara-C: aracytine; Dox: doxorubicin; PBS: phosphate-buffered saline. CFU: Colony forming unit. *p* < .05 is considered significant, n.s., not significant. *p < .05; **p < .01; ***p < .001.

After the *S. Typhimurium* challenge, we observed less translocations in Tg compared with Wt mice in both the liver (1.55 [1.33–2.19] log CFU/g vs. 2.27 [1.51–3.59] log CFU/g; *p* = .033) and the spleen (2 [1.85–2.2] log CFU/g vs. 2.94 [1.94–5.18] log CFU/g; *p* = .046). In control mice, which received PBS instead of chemotherapy, we did not observe significant translocation due to *S*. Typhimurium (Figure 7a-b).

## Discussion

To understand how the association of induction chemotherapy and antibiotics may affect the intestinal barrier and modify the intestinal microbiota, we prospectively monitored a cohort of patients with AML before, during, and after a 7 + 3 regimen. As expected, more than 70% of AML patients were in remission with the 7 + 3 regimen. Following chemotherapy administration, the patients lost weight, developed aplastic fever requiring broad-spectrum antibiotics, and were susceptible to *E. coli* septicemia. During the aplastic and recovery phases, we evidenced intestinal barrier impairment, as determined by citrulline levels, feces SCFA collapse, and deep modifications of intestinal microbiota combining a dramatic loss of overall bacterial load and alpha and beta diversities with a switch from anaerobic to aerotolerant bacteria.

As already observed, the overall decrease of bacterial populations was mostly due to the loss of oxygen-sensitive commensals such as *Clostridiales*. in favor of the relative abundance of *E. coli* and *Enterococci*.^4,14^ The quantitative stability of these bacteria in qPCR is consistent with the qualitative clustering of the samples in different patterns. In our study, *Lachnospiraceae* and *Ruminococcaceae*, which usually constitute a portion of the autochthonous human intestinal microbiota, were particularly affected, with a gradual relative enrichment of *Enterococcaceae, Enterobacteriaceae*, and *Pseudomonadaceae* that are non-dominant species in enterotypes of a healthy human.^15,16^ Although the significance of this observation on disease prognosis remains to be determined, this relative enrichment in aerotolerant bacteria might be associated with the increased risk of BSI caused by these pathogens during aplasia.

These gut microbiota modifications were concomitant with a decrease of citrulline and fecal SCFA levels, two surrogate markers of the functional enterocyte mass, and the microbial fermentative activity, respectively. Citrulline is a nonproteic amino acid produced mainly by enterocytes from glutamine. It was initially monitored in patients with short bowel syndrome, reflecting the reduction of functional enterocyte mass.^17,18^ In patients with hematological malignancies, it was highly correlated with the intestinal permeability test and the incidence of bacteriemia reflecting the intestinal impairment due to high-dose chemotherapy regimen.^19–22^ SCFA are produced mainly by colonic anaerobic bacteria from the fermentation of indigestible polysaccharides.^23^ SCFA has a trophic effect on colonocytes, regulating crypt depth, mucus secretion, and limiting the luminal expansion of bacteria.^24^ They also play a role in tissue homeostasis and their decrease limits intestinal repair (especially during graft versus host disease, GVHD) and colonic regulatory T-cell expansion.^25,26^

To examine the specific role of induction chemotherapy on the intestinal barrier, we mimicked AML induction in a mouse model without antibiotic administration. Our model was fairly representative of the effect of a 7 + 3 regimen in humans featuring transient weight loss, decrease of all blood cells, and loss of enterocyte functional mass as demonstrated by citrulline decrease and histologically an impairment of the terminal ileum. As opposed to what was observed in humans, changes in intestinal microbiota consecutive to chemotherapy in Wt mice, in the absence of antibiotic pressure, were mainly qualitative.^27^ Indeed, qPCR analyses did not reveal any loss of most bacterial populations, but compared with the baseline, *E. coli*, and *Enterococcus* spp. colonized the terminal ileum and translocated through the damaged intestinal epithelium. However, a comparison between human and mouse microbiota must be interpreted with caution because of difference in sample origin (feces *vs*. ileal adherent flora) and the different microbial ecology between human and mouse. It is noteworthy that microbiota diversity followed the same trend in human and Wt mice samples.

Next, we investigated the interest of mucosal strengthening in Tg mice. At baseline, a strengthening of the mucosal barrier in Tg mice was suggested by higher citrulline levels than those in Wt mice. While intestinal damage induced by chemotherapy was initially similar in both Tg and Wt mice, Tg mice had faster ileum repair three days after chemotherapy achievement, with lower tissue damage score, higher PCNA staining, and citrulline levels. The ileum microbiota of Tg mice was maintained while the microbial composition significantly changed after chemotherapy compared with Wt mice. Moreover, Tg mice appeared less sensitive to the *S. Typhimurium* translocation. Thus, we hypothesized that BSI proceeded in two steps: first chemotherapy-induced dysbiosis and an increase in the burden of aerotolerant bacteria and subsequently translocation of bacteria through the damaged intestinal barrier.^28^ Although baseline colonization was similar in Wt and Tg mice, our results show that mucus reinforcement could limit microbiota diversity impairment and pathological bacterial translocation after induction chemotherapy.^29^

For decades, improving the intestinal barrier has remained a challenge in patients undergoing chemotherapy. Various products have been evaluated to maintain intestinal homeostasis during the allo-SCT procedure, such as keratinocyte growth factor or analog of R-Spondin that inhibits heat shock proteins.^30–32^ In the last decade, our team demonstrated the beneficial role of early enteral nutrition, presumably due to its intestinal trophic effect on both GvHD severity and mortality in patients undergoing allo-SCT.^33–36^ More recently, we reported that increased macrophage reactivity and lower citrulline concentration before allo-SCT were strongly correlated with the incidence of GvHD in humans. These two parameters reflect persistent subclinical damage secondary to high-dose chemotherapies delivered during a 7 + 3 regimen.^37^ In addition, the interest of citrulline level as a strong predictive factor of GvHD before an allo-SCT procedure was recently confirmed.^38^ In our experience, citrulline level below 26 µmol/L before an allo-SCT constitutes an independent risk factor of severe gastrointestinal GvHD.^12^ Altogether, these data support the concept that maintaining intestinal integrity in patients receiving an AML induction regimen and further chemotherapies could limit microbiota dysbiosis responsible for infectious disease and further complications such as GvHD after allo-SCT.^39,40^

To our knowledge, this is the first translational study showing the deep modification of the intestinal barrier and physiopathology of BSI occurring after a 7 + 3 regimen. Our human study revealed the deep impairment of the intestinal barrier with a transient epithelium damage associated with a prolonged loss of load, diversity, and function of the microbiota. Our murine model determined more precisely the specific impact of chemotherapy, which is characterized by a qualitative dysbiosis and physical barrier impairment that facilitates bacterial translocation. As a proof of concept, we finally showed that strengthening the mucus can improve intestinal repair and maintain microbiota diversity thus limiting the risk translocation with entero-invasive bacteria.^41^ Although we need to determine if this protection is provided by the mucosal strengthening, change in microbiota, or reciprocal interactions, these results should lead to the development of new approaches to limit BSI and improve the outcome of patients with AML.

## Material and methods

### Human

#### Enrollment

This monocentric prospective observational study was conducted in the hematological ward of Gustave-Roussy Hospital between April 2013 and January 2014. Fifteen consecutive patients with AML received a conventional 7 + 3 regimen combining high-dose aracytine (cytarabine 200 mg/m^2^) for seven days and an anthracycline (idarubicin 12 mg/m^2^ or daunorubicin 60 mg/m^2^) for three days. Broad-spectrum antibiotics were administered in case of neutropenic fever, at the discretion of the clinician. Serum and feces were collected before induction (T0), during aplasia (T1) and after recovery phase (T2) were stored at −20°C and −80°C, respectively. At each time, clinical parameters (temperature, antibiotic intake, febrile episodes, and body mass index), biological parameters (disease status, aplasia, bacteriological documentation), and fecal analyses were recorded. Our institutional ethics committee approved the study and all patients signed a nonopposition form for the use of their data for the purpose of the study.

#### Fermentative activity

Concentrations of SCFA (acetate, propionate, butyrate, valerate, caproate, isocaproate, and isovalerate) in the feces were analyzed using gas-liquid chromatograph (Nelson 1020; Perkin-Elmer, St. Quentin en Yvelines, France) after water extraction of acidified samples, as described previously.^42^

### Mice

#### Induction chemotherapy model

Eight-to-10 weeks old female B6D2F1 wild-type (Wt) mice were used. Increased doses of aracytine (Accord Healthcare, France) and doxorubicin (Arrowlabs, India) were administered intraperitoneally for five and three consecutive days, respectively, as previously described.^43,44^ Increased doses from 50 to 150 mg/kg/d of aracytine and 1 to 3 mg/kg/d of doxorubicin were myelosuppressive and well tolerated. Doses over 150 mg/kg/d and 3 mg/kg/d, respectively, were deleterious with high rate mortality. Thus, we elected a regimen based on aracytine 150 mg/kg/d and doxorubicin 3 mg/kg/d. Mice were kept in the specific pathogen-free animal facility of the University of Lille. Housing conditions fulfilled the European guidelines for animal welfare. Weight and tolerance were evaluated each day until the sacrifice at one (d + 1), three (d + 3), and five (d + 5) days after the end of chemotherapy. Animal Care Committee of the region Nord–Pas de Calais approved all of the experimental protocols (APAFIS#8328-201622316064271v3).

#### Reinforced mucus layer model

We used transgenic mice (Tg222) releasing a recombinant molecule of 12 consecutive CYS domains (rCYSx12) GFP-tagged in their intestinal lumen. To generate the transgene and Tg222 mice, a Transgenic (Tg) plasmid containing the trefoil factor 3 (Tff3) promoter and an artificial exon encoding 12 CYS sequences was created. Then, the linearized DNA fragment was purified and injected into 4-week-old mice of a C57BL/6 genetic background. These modifications are associated with a reduced susceptibility to chemical-induced colitis and a reduced bacterial translocation after oral gavage of *Citrobacter rodentium*.^10^

Heterozygous transgenic female with a C57BL/6 genetic background was bred with DBA/2 Wt male mice, and pairs of cohoused female Tg and Wt B6D2F1 mice from the same litter were used throughout the present study. Four-week-old mice were screened for the presence of the transgene by PCR analysis using tail DNA extracted using specific primers as previously described.^10^ The amplified products were subjected to electrophoresis on a 12% acrylamide/bis-acrylamide gel. The presence of the transgene was confirmed by epifluorescence microscopy of fresh ileum or colon and by PCR.

#### Blood analyses

Blood was collected by cardiac puncture. White cell, red cell, and platelet counts were performed on the hematological counter (BC-2800Vet, Mindray, Shenzhen, China).

#### Histology and immunochemistry

On the day of sacrifice, 5-μm-thick sections of terminal ileum were prepared. Alternate sections were stained with Alcian blue (AB)-periodic acid-Schiff (PAS) and hematoxylin and eosin (HE). Villus height and crypt depth were determined on 10 villi in three different sections. To count goblet cells, the total number of PAS-positive cells was determined in 10 longitudinally sectioned crypts of villi of the ileum per section. Intestinal damage was assessed using a validated score of colitis, taking into account the extent of inflammatory cell infiltrates, epithelial changes with goblet cell loss, and mucosal architecture with villous blunting.^45^

Sections were stained immunohistochemically with anti-proliferating cell nuclear antigen (PCNA) using the PC10 anti-PCNA monoclonal antibody (Abcam, Cambridge, UK), as described previously.^10^ To assess apoptosis, we used a TDT-mediated dUTP-biotin nick end-labeling (TUNEL) method (Roche, Boulogne-Billancourt, France). When using the ab290 antibodies and TUNEL, a heat-mediated antigen retrieval step was performed before conducting the immunohistology.^46^ The sections used in immunofluorescence experiments were counterstained with Hoechst 33258 (1: 1.000)

#### Bacterial culture

Tissues (ileum, spleen, and liver) and feces were harvested and introduced into preweighed vials containing 1.5 mL of cysteinated Ringer’s solution. After physical disruption, dilutions were assessed and cultivable microbiota from tissues and feces were quantified.^46^ Total counts were conducted and different types of colonies were subcultured and identified according to established morphological and biochemical criteria. Final identification was confirmed by mass spectrometry (MALDI-TOFF, Biotyper instrument, Bruker Daltonics). *Salmonella Typhimurium challenge*

To evaluate the potential impact of mucus reinforcement, we studied intestinal translocation after a bacterial challenge by oral gavage the last day of induction chemotherapy. Thus, mice were infected with 100 μL of an overnight culture of Luria broth (LB) containing approximately 10^7^ CFU of the kanamycin-resistant GFP-tagged *Salmonella enterica* serovar Typhimurium (*S*. Typhimurium) strain and killed 2-day postinfection corresponding to d + 2. Feces and biopsy specimens of ileum, spleen, and liver were collected in a preweighed 2.0 mL microtube containing 1.0 mL of PBS. Tissue and feces were weighed and then homogenized with a pellet pestle. Tissues and feces were serially diluted in phosphate buffer saline (PBS), plated onto LB agar plates containing 100 mg/mL of kanamycin sulfate (Sigma-Aldrich, Saint Louis, MO) and incubated overnight at 37°C.^47,48^ *S*. Typhimurium colonies were counted the following day and normalized to tissue weight.

### Human and mouse

#### Plasma citrulline level

Fasting plasma from human and mouse was deproteinized with a sulfosalicylic acid solution and the supernatants were stored at −80°C until analysis. Plasma citrulline concentrations were assessed by high-performance liquid chromatography combined with tandem mass spectrometry as previously described.^49^

#### DNA extraction and qPCR of microbiota in human feces and in mouse terminal ileum

Total DNA was extracted from aliquots of 200–250 mg of human feces and 12 to 28 mg of mouse terminal ileum without stool according to the protocol described by Godon et al.^50^ Dominant bacteria groups such as aerotolerant (*Colibacillus, Lactobacillus, Streptococcus, Enterococcus*) and anaerobic bacteria (*Bifidobacterium, Bacteroides, Clostridium leptum*/*coccoides, Akkermansia*) present in the samples at each time was evaluated using quantitative PCR (qPCR) analyses, as previously described.^51^ PCR inhibition was tested with fecal and ileum mucosal DNA dilutions using a TaqMan exogenous internal positive control (Applied Biosystems, Carlsbad, CA). No inhibition was detected using 10^−3^ dilutions for fecal DNA and 8 10^−2^ dilutions for ileal DNA; consequently, these dilutions were used for all PCR amplifications.

#### Microbiota characterization in human feces and in mouse terminal ileum

Bacterial diversity was assessed for each sample by targeting the V3 and V4 hypervariable regions of the 16S ribosomal RNA-coding gene and was amplified with the primers‘16SFor’ 5ʹ- CTTTCCCTACACGACGCTCTTCCGATCTTACGGRAGGCAGCAG −3ʹ and ‘16SRev’ 5ʹ- GGAGTTCAGACGTGTGCTCTTCCGATCTTACCAGGGTATCTAATCCT −3ʹ). This first PCR reaction was performed using 10 ng of DNA, 0.5 μM primers, 0.2 mM dNTP, and 0.5 U of the DNA-free MOLTaq 16S DNA-polymerase (Molzym), using the following PCR profile: 1 cycle at 94°C for 60 s, followed by 30 cycles at 94°C for 60 s, 65°C for 60 s, 72°C for 60 s, with an end-step at 72°C for 10 min. The PCR reactions were sent to GenoScreen (Lille, France) for sequencing using Illumina MiSeq technology. Single multiplexing was performed using an optimized and standardized 16S-amplicon-library preparation protocol (Metabiote; GenoScreen, Lille, France). Briefly, 16S rRNA gene PCR was conducted using 5 ng of genomic DNA according to Metabiote protocole instructions using 192 bar-coded primers (Metabiote MiSeq Primers; GenoScreen) at final concentrations of 0.2 μM and an annealing temperature of 50°C for 30 cycles. PCR products were cleaned up using an Agencourt AMPure XP-PCR Purification system (Beckman Coulter, Brea, USA), quantified according to the manufacturer’s protocol, and multiplexed at equal concentration. Sequencing was performed using a 300-bp paired-end sequencing protocol on an Illumina MiSeq platform (Illumina, San Diego, CA) at GenoScreen. The next steps were realized with Snakemake to manage QIIME (2019.1) software.^52,53^

#### Bioinformatic analyses

Sequences were joined and then filtered.^54^ Operational Taxonomic Units (OTUs) were created, with Deblur software, truncating the sequence at 300 bp.^55^ Blast+ and SILVA database (release 128, version 99) were used to annotate sequences.^56,57^ Then, Mafft aligned sequences and phylogenetic trees were built with FastTree to calculated diversity metrics.^58,59^

Microbiome diversity was analyzed using alpha and beta-diversity metrics. Alpha-diversity, representing number and abundance of species within samples, was studied using Shannon and Chao-1 indices. Beta-diversity was estimated using unweighted Unifrac distances representing OTU presence or absence.^60,61^ Diversity analysis was assessed with 10360 and 9000 sequences for the human and the murine studies, respectively. A PERMANOVA test was used to compare beta-diversity between groups. Principal coordinate analysis (PCoA) of unweighted Unifrac distances was conducted with Emperor to study the evolution of beta-diversity over time.^62^

### Statistical analyses

Continuous variables were presented as median and range or interquartiles range. For the human study, the non-parametric Friedman test was used. Comparisons for each patient between T0 and T1, and T0 and T2 were conducted using the Wilcoxon signed-rank test. One patient who died between T0 and T1 was excluded from the analysis.

For mouse experiments, the Kruskal-Wallis with a posthoc Dunn’s correction was used to conduct multiple comparisons. The Wilcoxon signed-rank test was used to compare cohoused Wt and Tg mice from the same litter. Overall survival was estimated using a log-rank test and represented by Kaplan–Meier curves.

Statistical significance was defined as *p* < .05 based on two-sided tests. Statistical analyses were performed using R, version 3.1.0 (R Foundation for Statistical Computing) and GraphPad Prism, version 6.0 (GraphPad Software, La Jolla, CA).

## Supplementary Material

Supplemental Material
