# Supplementary material for: Impact and consequences of intensive chemotherapy on intestinal barrier and microbiota in acute myeloid leukemia: the role of mucosal strengthening
Source: Gut Microbes. 2020 Sep 6;12(1):1800897. doi: 10.1080/19490976.2020.1800897 (PMC7524297; doi:10.1080/19490976.2020.1800897)
Supplement: Supplemental Material [file KGMI_A_1800897_SM9872.doc]

**Supplementary data**

**Figure S1**.

Unweighted Unifrac principal component analysis showed the clustering of the two groups of samples: T0, before induction (red circle); T1 and T2 during aplasia and after hematological recovery respectively (yellow circle)

**Figure S2.**

**a.** Bacterial colonization in terminal ileum and translocation in the spleen and liver at baseline (Bsl) and d+3, after completion of induction chemotherapy in Wt mice **b.** Mass spectrometry (MALDI-TOFF) identified exclusively *Lactobacillus* spp. *(*green circle*)* at Bsl and also *Escherichia* *coli* (blue circle) and/or *Enterococcus* spp. (orange circle) at d+3 in the ileum, spleen and liver. Bsl: Baseline; d+3: three days after chemotherapy completion; CFU: colony form unit; n.s.: not significant.

**Figure S3.**

Quantitative qPCR analyses of adherent ileal mcrobiota in Tg222 and Wt mice at d+3 depicted higher concentration in clostridiales in Tg222 with similar concentration in Lactobacillus. AraC: Aracytine; Dox: Doxorubicin; Ctrl : Control mice. *p* < 0.05 was considered significant.

**Figure S4.**

Unweighted Unifrac principal component analysis. Control mice with Tg222 (red circle) and Wt mice (blue circle) are represented and clustered in the red circle. Three days after chemotherapy completion, Tg222 (red square) and Wt mice (blue square) are clustered in the yellow circle.


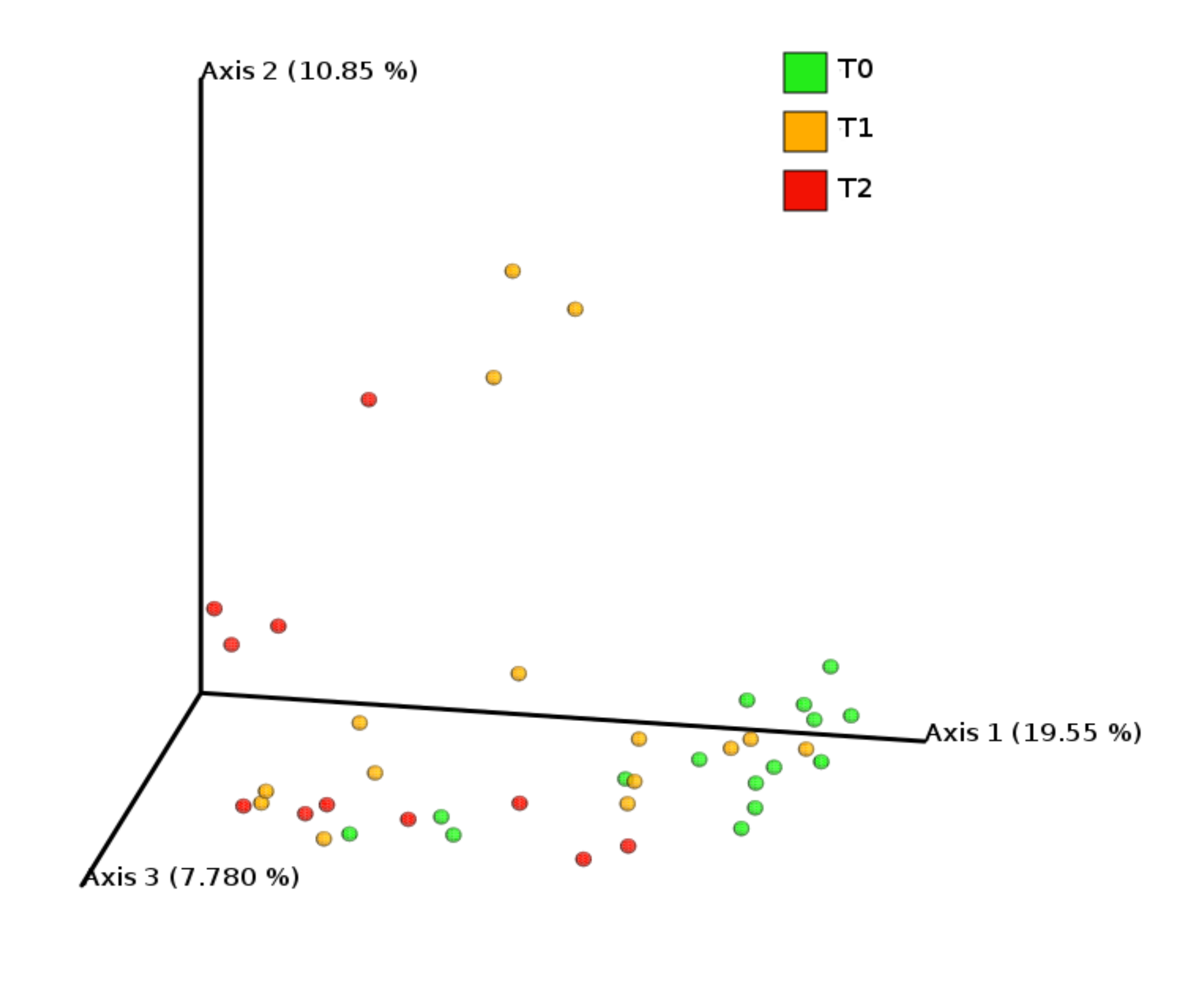


**Figure S1**.

**a.** **b.**

**
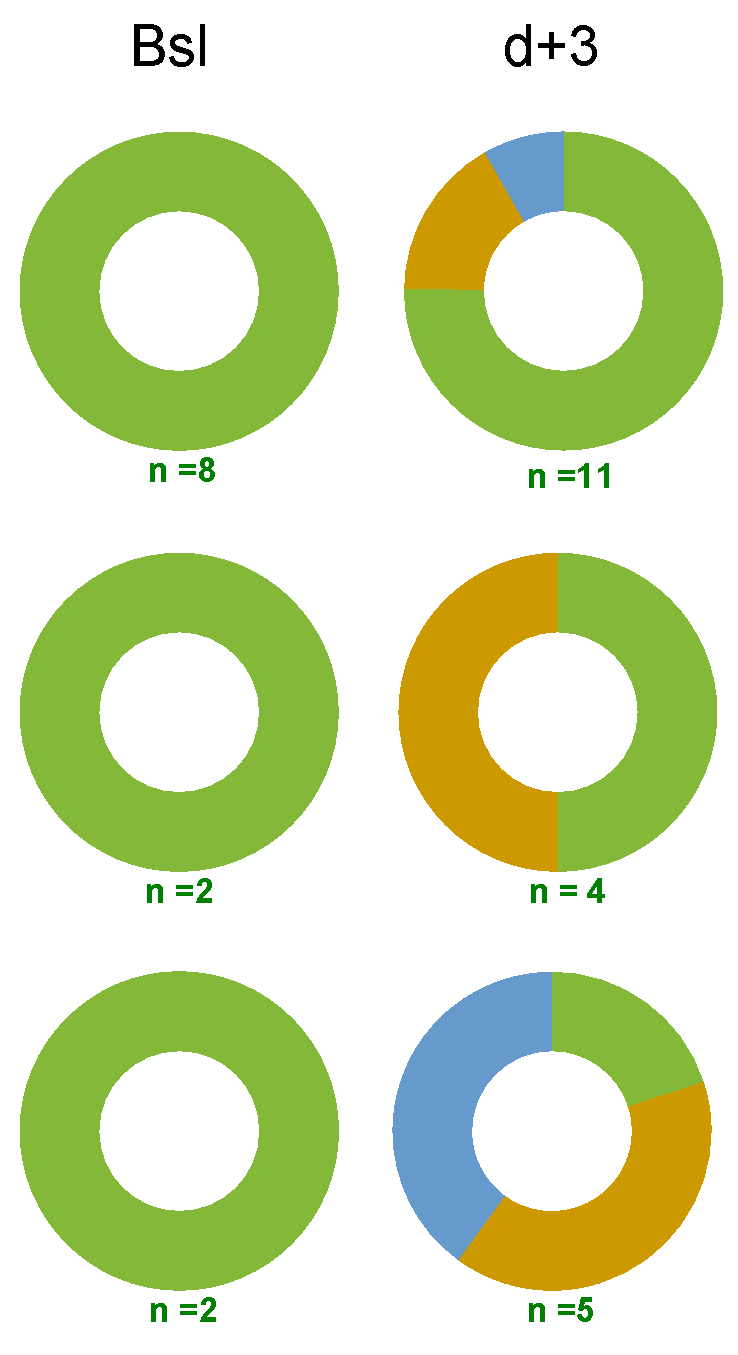
**

**
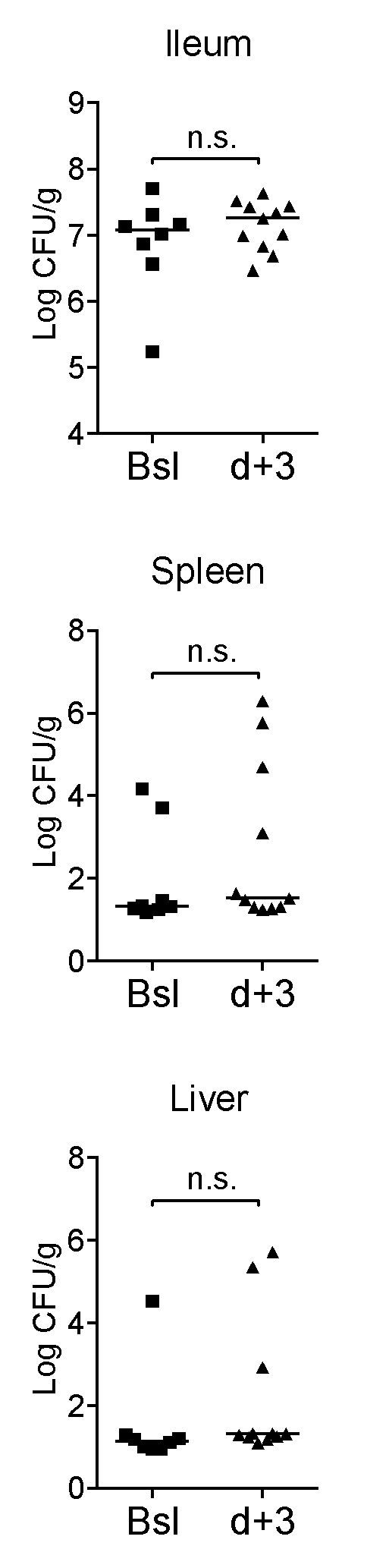
**

**Figure S2.**

**Figure S3.**

**
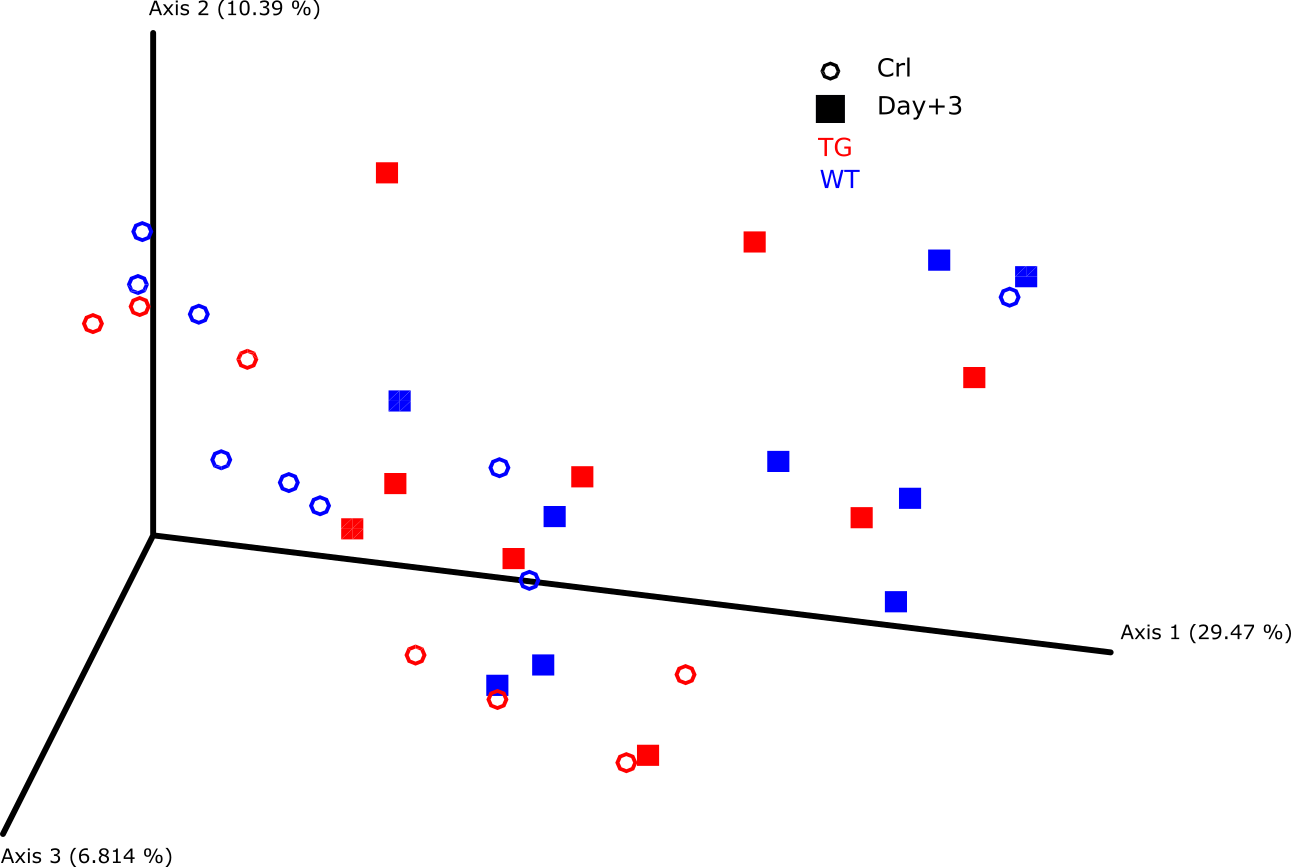
**

**Figure S4.**
